# Interleukin-33 at the intersection of inflammation and repair

**DOI:** 10.3389/falgy.2026.1894127

**Published:** 2026-07-16

**Authors:** Fernanda Martinez-Moreno, Elina Jerschow, Victor E. Ortega, Hirohito Kita, Sergio E. Chiarella

**Affiliations:** 1 Division of Pulmonary, Critical Care, Allergy, and Sleep Medicine, Mayo Clinic, Rochester, MN, United States; 2 Division of Pulmonary and Critical Care Medicine, Mayo Clinic, Scottsdale, AZ, United States; 3 Division of Allergy, Asthma and Clinical Immunology, Mayo Clinic, Scottsdale, AZ, United States

**Keywords:** alarmin, epithelial repair, interleukin-33, nuclear sequestration, protease cleavage, redox regulation, therapeutic strategies, type 2 inflammation

## Abstract

Interleukin 33 (IL-33) is a cytokine of the IL-1 family that acts as an alarmin in both innate and adaptive immunity. IL-33 is constitutively nuclear in epithelial and endothelial cells, where its activity is limited by nuclear retention. Release of IL-33 in response to cellular stress or injury can mediate type 2 immune responses or tissue repair, depending on the local tissue environment. IL-33 activity is regulated at multiple levels, including nuclear retention, oxidation, proteolytic processing, and metabolic regulation. Following its release, oxidation rapidly suppresses IL-33 activity, while mast cell and neutrophil proteases can generate smaller active fragments that target cells expressing the IL-33 ST2 receptor. Simultaneously, tissue metabolic status influences cellular responsiveness through the mTORC1 and AMPK pathways, which link metabolic capacity to effector function. These conditions may explain why IL-33 can mediate an inflammatory response, but in other circumstances, it contributes to tissue repair. Such pleiotropic properties may also underline the variability in clinical responses to IL-33/ST2-targeted therapies across various diseases. The appreciation of IL-33 as a cytokine whose activity is conditioned by its structural, redox, and metabolic environment is critical to optimizing its therapeutic potential as a target.

## 1 Introduction

Interleukin-33 (IL-33) belongs to the IL-1 cytokine family, but it does not behave like a typical secreted cytokine. A defining feature of IL-33 is that its biology starts inside the cell. Under homeostatic conditions, IL-33 is retained in the nuclei of epithelial and endothelial cells, where its interaction with chromatin limits premature extracellular activity (1–8). In this state, IL-33 is already present, but it is not yet acting as an extracellular signal. We define this intracellular stage as a pre-alarmin phase, where IL-33 is stored, restrained, and ready to be released upon cell injury or stress (2, 4, 6). When epithelial barriers are disrupted, or when cells undergo stress or damage, IL-33 can leave this nuclear compartment and act on nearby immune and stromal cells (6, 7, 9–13).

Once IL-33 is released, its effect is not fixed. In some settings, IL-33 amplifies inflammation by activating ST2-expressing cells, including ILC2s, mast cells, eosinophils, and other immune populations. This response can promote type 2 immunity and contribute to the development of allergic and inflammatory diseases (1, 9, 10, 14–19). In other settings, IL-33 supports tissue adaptation, repair and restoration of barrier stability, partly via regenerative and regulatory pathways linked to amphiregulin (7, 20–27). Thus, the key question is not simply whether IL-33 is released. The more important question is what kind of IL-33 signal is generated, in which tissue, and under what conditions (6, 7, 13, 28–32).

Several checkpoints modulate this signal. Nuclear retention regulates the timing of IL-33 release. After release, IL-33 activity is further shaped by proteolytic processing, oxidative inactivation, receptor availability, and the redox and metabolic state of the surrounding tissue (6, 13, 28–30, 33–36). These results indicate that IL-33 is not simply an alarm that is on or off. Rather, IL-33 behaves as a context-sensitive signal whose meaning changes with the state of the tissue, particularly during stress, injury, inflammation and repair (7, 13, 28, 29, 31, 32, 36).

In this review, we follow IL-33 from nuclear retention to extracellular signaling. We discuss how intracellular sequestration, release, post-release processing, and metabolic state influence whether IL-33 promotes inflammation, supports tissue repair, or does both in sequence. We also discuss whether these checkpoints can be therapeutically used to limit pathogenic IL-33 activity while preserving its beneficial roles in homeostasis and repair.

## 2 Nuclear retention as a molecular checkpoint in the pre-alarmin phase

Although IL-33 is widely described as an alarmin, its biology begins before release. Under homeostatic conditions, IL-33 is not mainly a secreted cytokine. It is a nuclear protein, a feature recognized after its identification as a ligand for ST2 (1, 2, 4). In epithelial and endothelial cells, IL-33 is constitutively retained in the nucleus, forming a stable intracellular reservoir enriched in barrier tissues such as the lung, skin, and intestine (2–4, 7). This arrangement allows barrier tissues to maintain IL-33 in a ready state for rapid release after injury, while limiting inappropriate signaling under basal conditions.

Structurally, IL-33 is a 30 kDa protein with an N-terminal domain involved in nuclear localization and chromatin binding, and a C-terminal IL-1-like cytokine domain (4, 37). The N-terminal region contains a helix-turn-helix motif that interacts with histones H2A and H2B, anchoring IL-33 to chromatin and limiting its diffusion into the cytoplasm (4, 5, 8). This chromatin association helps prevent constitutive release and unintended activation of ST2-expressing cells. *In vivo*, the importance of this nuclear restriction was shown by Bessa *et al*., who reported that impaired nuclear localization of IL-33 led to systemic, non-resolving inflammation, supporting the idea that nuclear retention is required for immune homeostasis (38).

Beyond this restraining function, several studies suggest that nuclear IL-33 can also have cell-intrinsic regulatory roles. Full-length IL-33 (IL-33_FL_) has been shown to interact with NF-κB and dampen NF-κB-driven transcription, suggesting that nuclear IL-33 may help restrain inflammatory gene expression in some contexts (39). In endothelial cells, nuclear IL-33 has also been reported to regulate NF-κB p65 and contribute to endothelial activation (37). Other studies have extended this idea beyond endothelial biology. Nuclear IL-33 has been linked to SMAD signaling in epithelial cells during chronic inflammation-associated cancer, and epidermal nuclear IL-33 has been implicated in keratinocyte migration and wound closure through STAT3 and NF-κB pathways (40, 41). These findings suggest that nuclear IL-33 may participate in transcriptional or stress-adaptive programs, particularly in settings of epithelial injury, chronic inflammation, or repair.

At the same time, this nuclear function should not be overstated. Gautier *et al*. found that endogenous nuclear IL-33 did not reproducibly regulate the endothelial proteome, arguing that, at least in endothelial cells, the dominant role of nuclear localization may be to restrain extracellular cytokine activity rather than to act as a broad transcriptional regulator (8).

Several factors may explain these apparently divergent findings. One possibility is that nuclear IL-33 does not have the same role in every cell type. In endothelial cells under basal or near-homeostatic conditions, nuclear retention may mainly serve to keep IL-33 sequestered and prevent premature extracellular cytokine activity, as suggested by studies in endothelial cells showing limited global proteomic effects of endogenous nuclear IL-33 (8). In contrast, epithelial cells exposed to chronic inflammation, injury, or wound-repair signals may be more likely to reveal transcriptional or chromatin-associated functions, including SMAD-related signaling in inflammation-associated epithelial cancer and STAT3/NF-κB-dependent keratinocyte migration during wound closure (40, 41). The experimental setting is also important. Overexpression systems, inflammatory stimulation, cancer-associated inflammation, and wound-healing models may uncover activities that are not evident under basal conditions (37, 39–41). Finally, different studies have used different readouts, from targeted transcriptional assays and pathway-specific measurements to broader proteomic approaches (8, 37, 39–41).

The nuclear compartment also biochemically protects IL-33. Outside the cell, oxidative modifications can quickly inactivate IL-33. In the nucleus, reducing conditions, by contrast, help preserve IL-33 in a form that remains competent for cytokine activity once released (13, 28). Chromatin binding regulates IL-33 release and extracellular activity, supporting the idea that nuclear retention is not passive storage, but a functional checkpoint in the IL-33 pathway (6).

Therefore, we consider nuclear IL-33 to represent a pre-alarmin phase. In this phase, IL-33 is present and stable, but not yet available for extracellular signaling. It can be mobilized quickly if the cell membrane integrity is disturbed. This makes nuclear retention more than a localization feature. It determines when IL-33 can act, how strong the downstream signal may become, and which type of response is likely to follow. We use this term as a conceptual framework rather than as a formally established biochemical state, to distinguish nuclear IL-33 from its extracellular alarmin activity.

## 3 Functional outcomes of IL-33 signaling

After release into the extracellular space, IL-33 binds ST2, which is expressed by several innate and adaptive immune cells, including ILC2s, mast cells, basophils, eosinophils, T cells, and regulatory T cells (Tregs) (1, 7, 42). In ILC2s and CD4⁺ T cells, ST2 signaling induces type 2 cytokines, including IL-5 and IL-13, as well as amphiregulin. These mediators promote eosinophil recruitment, mucus secretion, and early epithelial responses to injury (1, 7, 9, 10, 25).

In experimental injury models, IL-33-activated ILC2s promote epithelial restoration through amphiregulin-EGFR signaling. Thus, the same pathway that can increase inflammation of type 2 can also support barrier recovery, if the signal is transient and spatially restricted (25, 43). This duality is central to IL-33 biology, as the same molecule can drive inflammation and support tissue repair.

IL-33 further modulates adaptive immunity by promoting the expansion and functional adaptation of tissue-resident Tregs, which can produce IL-10 and amphiregulin, and contribute to immune regulation and tissue repair (26, 44, 45). This reparative function is not just a consequence of suppressing inflammation. Arpaia et al. have shown that inflammatory mediators, including IL-33, can induce a tissue-protective Treg program characterized by amphiregulin production, separating tissue repair from classical Treg immunosuppression (44). These results indicate that IL-33 links epithelial damage to immune programs that can amplify inflammation and promote recovery.

The problem is that these responses can change over time. In acute settings, IL-33 often helps protect and repair tissue by activating regulatory and reparative pathways. But if IL-33 continues to be released during ongoing epithelial stress or repeated injury, it can maintain activation of ILC2 and Th2 cells. This can drive chronic type 2 inflammation, airway remodeling, and fibrosis (17, 46, 47). This distinction is especially important in airway disease, where IL-33-expressing epithelial progenitor populations have been linked to both repair and remodeling in the distal airway. A program that normally supports epithelial renewal may therefore contribute to structural disease when it is repeatedly activated (48).

The direction of the IL-33 response appears to depend on how the signal is delivered and how long it persists. When IL-33 is released acutely and locally, in a tissue environment with limited protease amplification, intact redox control, and dominant ILC2/Treg-amphiregulin responses, the pathway is more likely to support epithelial repair and restoration of barrier integrity. In contrast, persistent IL-33 release, repeated epithelial injury, protease-rich allergen exposure, or sustained activation of ILC2/Th2-eosinophilic circuits can shift the response toward chronic type 2 inflammation, mucus production, remodeling, and fibrosis. Strong oxidative inactivation or increased decoy receptor activity may shorten this signaling window and limit both inflammatory and reparative programs. Thus, the balance between the responding cell type, signal duration, and local tissue conditions helps determine whether IL-33 functions as a reparative cue or as a pathological amplifier.

## 4 Intrinsic regulation

Intrinsic regulation describes how IL-33 moves from its nuclear pre-alarmin state to extracellular availability. This section is not about where IL-33 resides; that checkpoint has already been discussed. The question here is when, how, and in what form IL-33 leaves the cell. One level of control is between expression and release. Inflammatory cytokines such as IL-1β and TNF-α, as well as Toll-like receptor activation, can induce IL-33 transcription in fibroblasts and keratinocytes. However, increased expression of IL-33 does not necessarily mean secretion, because IL-33 remains restricted to the nucleus unless cell structural integrity is compromised (4, 49, 50). In this sense, IL-33 can accumulate in a primed, but restrained state, ready for rapid mobilization, but still silent as an extracellular cytokine.

During apoptosis, caspases 3 and 7 cleave IL-33 within its IL-1-like cytokine domain, generating fragments that cannot bind ST2 and are therefore biologically inactive (34, 51). This preserves the immunological silence normally associated with apoptotic cell death. Necrosis has the opposite effect. It releases full-length IL-33, which can still engage ST2 and activate NF-κB and MAPK signaling in nearby cells (1, 34).

IL-33 can also be mobilized through more regulated forms of cell stress. Epithelial cells can release IL-33 through caspase-8-dependent apoptotic signaling or through RIPK3/MLKL-mediated necroptosis, linking programmed cell death pathways to cytokine availability (12, 52). Non-lytic release has also been described in response to purinergic and cholinergic signals, especially during exposure to allergens and microorganisms (9, 53, 54).

Environment triggers add another layer. Aeroallergens such as fungal spores, pollen and house dust mite extracts are able to disrupt epithelial integrity and induce intracellular signaling events, including Ca^2+^ fluxes, that promote rapid IL-33 release from airway epithelial cells (9, 10, 30). Importantly, these stimuli mainly induce cell stress and cell release. They do not activate IL-33 directly.

Transcriptional priming, cell death pathways, and regulated secretion define the final checkpoint of the pre-alarmin phase and determine when IL-33 becomes available to the extracellular environment. These mechanisms establish an intrinsic regulatory network that controls IL-33 availability at the point of release (Table 1).

**Table 1:** Summary of modes of IL-33 regulation during cell stress and death.

Cell condition	Effects on IL-33	IL-33 outcome	Functional interpretation (12, 52, 54, 76)
Apoptosis	Caspase 3/7-mediated cleavage	Inactivation	Silent turnover
Necrosis/necroptosis	Nuclear rupture and extracellular release	Full-length release	Alarm signal
Ripoptosome activation	Caspase 8-mediated processing	Activation	Inflammation/immune response
Cell membrane perforation	Extracellular release	Full-length release	Inflammation/immune response
Inflammatory environment	Protease cleavage	Activation	Inflammation/immune response
Cellular activation	Protease cleavage	Activation	Inflammation/immune response
Oxidative environment	Disulfide bond formation	Inactivation	Resolution

## 5 Extrinsic regulation

After release, IL-33 enters a tissue environment that immediately begins to shape its activity. IL-33 does not simply diffuse as an unchanged signal. It encounters proteases, oxidants, receptors, and local biochemical conditions that determine how long it signals, how strongly it signals, and what type of response it supports (Table 1).

One major layer of extrinsic regulation is the proteolytic processing. IL-33_FL_ is cleaved by proteases from mast cells and neutrophils including chymase, cathepsin G and elastase. These enzymes produce truncated IL-33 forms with enhanced bioactivity (30, 33, 35). This processing does not initiate the IL-33 signaling, but it is able to amplify the signal after release. Protease-rich environments, such as those associated with allergen exposure, can thus turn IL-33 into a more potent inflammatory mediator.

This distinction helps explain why IL-33 can support repair in one setting and pathology in another. A transient release of IL-33_FL_ may support local epithelial recovery. In contrast, protease-rich allergic environments can convert the same alarmin signal into a stronger and sustained type 2 inflammatory program (30, 33, 35). In this sense, extracellular processing not only modifies IL-33. It alters the biological meaning of the signal.

IL-33 contains conserved cysteine residues that form rapid intramolecular disulfide bonds under oxidative conditions. This changes the IL-33 conformation and prevents ST2 binding (28). Redox-dependent inactivation thus acts like a molecular timer. It limits the duration of IL-33 activity and helps prevent excessive immune activation. Oxidation may help shut down the IL-33 activity after an acute injury response, once the need for repair has passed. Conversely, if proteolytic amplification outweighs redox inactivation, IL-33 signaling can go beyond the reparative window and contribute to remodeling.

Alongside membrane-bound ST2, a soluble form of the receptor, sST2, is generated by alternative splicing of IL1RL1. By binding extracellular IL-33, sST2 limits the bioavailability of IL-33 and dampens signaling (18, 42, 55). Importantly, sST2 is not only a passive circulating buffer. Its production can increase in specific inflammatory or stress-related contexts. Activated mast cells can synthesize and release sST2 after antigen- or IL-33-dependent stimulation, suggesting that local immune cells can actively limit IL-33 signaling during allergic inflammation (55). Mechanical stress can also induce the IL-33/ST2 axis in cardiac tissue, where sST2 functions as a soluble decoy receptor and reflects biomechanical strain (56). In the airways, elevated serum sST2 has been reported during acute asthma exacerbations, supporting its potential role as a marker of active tissue stress and inflammatory burden (57). In addition, vitamin D has been shown to enhance sST2 production and thereby inhibit IL-33 activity, suggesting that endocrine or metabolic signals may also regulate this decoy pathway (58). These findings indicate that sST2 may function both as a regulator of IL-33 bioavailability and as a biomarker of the tissue contexts in which the IL-33/ST2 axis is activated.

The balance between proteolytic activation, oxidative inactivation, and receptor availability determines the effective signaling window of IL-33. In protease-rich environments, IL-33 activity is amplified and sustained, favoring type 2 inflammation and tissue remodeling. In highly oxidative environments, such as those observed in chronic obstructive pulmonary disease or fibrotic tissues, IL-33 may be rapidly inactivated, limiting its capacity to support tissue repair (28). Thus, repair is not an automatic consequence of IL-33 signaling. It depends on the duration, intensity, and biochemical form of the extracellular IL-33 signal.

## 6 Metabolic control and stress integration

IL-33 is also linked to the metabolic and redox state of tissues. From nuclear retention to extracellular signaling, the activity of IL-33 is influenced by cellular energy balance, oxidative stress and metabolic adaptation (13, 28, 59). Oxidative conditions rapidly inactivate extracellular IL-33 through disulfide bond formation, while the reducing conditions within the nucleus help preserve IL-33 in a bioactive state before release (13, 28). These data place IL-33 at the intersection between tissue stress sensing and metabolic regulation.

IL-33 also promotes metabolic reprogramming in immune and stromal cells. In ILC2s, regulatory T cells, macrophages, and Th2-associated responses, IL-33 signaling supports glycolysis, lipid utilization, anabolic metabolism, cytokine production, cellular proliferation, and tissue adaptation (20, 21, 59). In ILC2s, IL-33 activates PI3K-AKT-mTORC1 signaling pathway, promoting glycolytic metabolism and effector cytokine production. Inhibition of mTORC1 reduces IL-13 secretion and limits ILC2 activation (21, 59, 60).

Epithelial restoration, amphiregulin production and immune cell expansion all require energy and biosynthetic resources. Thus, IL-33-driven repair is not only an immunologic program, but also a metabolically demanding process. IL-33 also contributes to metabolic adaptation in macrophages and stromal cells, connecting local immune activation to broader processes such as thermogenesis, adipose tissue remodeling and systemic energy balance (24, 27, 61, 62).

These responses appear to be limited by the cellular metabolic capacity. The mTORC1-AMPK axis integrates nutrient availability, ATP levels and oxidative stress, controlling the magnitude and persistence of IL-33-driven responses (20, 21, 59, 63). Under nutrient-rich conditions, mTORC1 supports anabolic metabolism and effector function downstream of ST2 activation. During ATP depletion or oxidative stress, AMPK activation suppresses NF-κB-dependent inflammatory programs and limits cytokine production (36). These results indicate that IL-33-induced inflammation is not only triggered by tissue damage. It is also constrained by the metabolic resources needed to sustain it. The same principle may apply to repair; acute IL-33 signaling may support restoration when resources are available, whereas metabolic stress may limit repair or redirect IL-33 responses toward persistent inflammation and remodeling.

Extracellular IL-33 promotes anabolic activation and immune-cell expansion. Nuclear IL-33, in contrast, has been associated with chromatin-related regulatory programs linked to cellular equilibrium and stress adaptation (37, 40, 64). Loss of nuclear IL-33 has been associated with dysregulated mTORC1 signaling and chronic metabolic inflammation, suggesting that nuclear sequestration may restrain some of the same inflammatory and anabolic pathways activated by extracellular IL-33 (8, 40). Redox status also regulates IL-33 across compartments: high levels of reactive oxygen species rapidly oxidize and inactivate extracellular IL-33, whereas mild oxidative stress within the nucleus has been linked to increased transcriptional activity and stress-responsive signaling (13, 28, 64).

Taken together, these metabolic and redox checkpoints suggest that IL-33 is more than a warning signal. IL-33 signaling appears integrated with the energetic and oxidative conditions of the surrounding tissue. This suggests that IL-33 acts as a regulator of inflammatory permissiveness, promoting immune activation when adequate metabolic resources are available and restricting prolonged responses under energetic or oxidative stress. In this system, repair may represent the most balanced outcome of IL-33 signaling, enough activation to restore tissue stability, but also metabolic and redox restraint to avoid chronic remodeling.

Although IL-33-driven metabolic reprogramming has been linked to pathways such as mTORC1 and AMPK, this remains an emerging area of study (20, 21, 59, 60). Current evidence shows that IL-33 can reshape metabolic programs in responding immune and stromal cells, and that the metabolic state of these cells influences the magnitude of IL-33-driven responses. However, these findings do not yet demonstrate that IL-33 directly senses cellular energy status. At present, IL-33 is better viewed as a cytokine whose activity is conditioned by the tissue's metabolic state, rather than as a bona fide metabolic sensor. Determining whether IL-33 directly interacts with metabolic sensing pathways or instead acts indirectly through ST2-dependent activation of metabolically responsive cells will require metabolic flux analyses, redox-sensitive signaling studies, and epigenetic characterization of nuclear IL-33 functions. Resolving this question may help explain why IL-33 promotes tissue repair in some settings, while sustaining chronic inflammation in others.

## 7 Clinical modulation of the IL-33/ST2 pathway in inflammation and repair

The clinical experience with IL-33/ST2 blockade has been useful, though it has not yielded a simple answer. IL-33 is an appealing therapeutic target because it sits upstream of several inflammatory pathways, especially in barrier tissues exposed to allergens, smoke, infection, or repeated injury (Table 2). At the same time, the biology discussed above argues against a purely suppressive view of IL-33. This pathway can amplify pathogenic inflammation but also contribute to tissue adaptation and repair. The therapeutic challenge is therefore not only whether IL-33 can be blocked. The harder question is whether its harmful activity can be limited without interfering with short-lived repair programs that follow acute injury.

**Table 2:** Clinical studies targeting the IL-33/ST2 pathway.

Author, year, target	Clinical model		Main results	
Chen et al. (65)	Moderate-to-severe atopic dermatitis	Proof-of-concept clinical trial using a single dose of etokimab	Rapid clinical improvement was reported, with 83% achieving EASI50 and 33% achieving EASI75; skin and peripheral inflammatory markers also decreased.	IL-33 can contribute to human atopic inflammation, but this does not prove that chronic disease can be controlled by IL-33 blockade alone
Etokimab; anti-IL-33				
Chinthrajah et al. (66)	Peanut allergy	Phase 2a randomized, placebo-controlled study; single-dose etokimab followed by oral food challenges	At day 15, 73% of etokimab-treated participants tolerated 275 mg peanut protein vs. 0% placebo; Th2 cytokines and peanut-specific IgE were reduced.	IL-33 may be especially relevant in acute allergen-driven responses
Etokimab; anti-IL-33				
Wechsler et al. (69)	Moderate-to-severe asthma	Randomized trial testing itepekimab alone and in combination with dupilumab	Itepekimab monotherapy reduced loss-of-asthma-control events and improved lung function and quality-of-life measures.	IL-33 can drive clinically meaningful airway inflammation, but patient selection is likely important
Itepekimab; anti-IL-33				
Kelsen et al. (70)	Severe uncontrolled asthma	Phase 2 randomized placebo-controlled trial; astegolimab at different doses	Astegolimab reduced annualized asthma exacerbation rate, including in eosinophil-low patients.	IL-33/ST2 biology may extend across type 2-high and some type 2-low asthma phenotypes
Astegolimab; anti-ST2				
Crim et al. (77)	Moderate-to-severe uncontrolled asthma	Multicenter phase 2A trial of IL-33 receptor inhibition	The study reported reduced loss of asthma control with GSK3772847 vs. placebo, suggesting possible benefit.	Blocking ST2 can affect asthma outcomes, but pathway engagement does not guarantee a robust clinical effect
GSK3772847/melrilimab; anti-IL-33 receptor/ST2				
Akinseye et al. (78)	Moderate-to-severe asthma with allergic fungal airway disease	Phase 2a randomized multicenter trial	The study was terminated early due to high screen failure rates and low enrollment; no clear clinical benefit was observed.	Even rational phenotypes may fail if the pathway is not active enough, or if recruitment and sample size limit interpretation
GSK3772847/melrilimab; anti-IL-33 receptor/ST2				
Rabe et al. (72)	Moderate-to-severe COPD	Genetic association study plus randomized phase 2a trial	Primary endpoint was not met in the overall COPD population; former smokers showed reduced exacerbations and improved FEV1, while current smokers did not benefit.	Tissue context matters. Active smoking and redox stress may change whether IL-33 blockade works
Itepekimab; anti-IL-33				
Yousuf et al. (73)	Moderate-to-very severe COPD	COPD-ST2OP phase 2a placebo-controlled trial	Astegolimab did not significantly reduce exacerbation rate but improved health status compared with placebo.	ST2 blockade may modify COPD biology, but may not be sufficient to reduce exacerbations in broad COPD populations
Astegolimab; anti-ST2				
Rabe et al. (79)	COPD in former smokers	AERIFY-1/2 phase 3 trial design paper	Described two phase 3 trials designed to test whether the phase 2 former-smoker signal can be confirmed.	Clinical development is already testing the idea that IL-33 blockade needs the right biological context
Itepekimab; anti-IL-33				
Singh et al. (74)	COPD with chronic bronchitis	FRONTIER-4 phase 2a signal-finding study	The primary endpoint was not met in the overall population, but signals suggested a possible benefit in high-risk or exacerbation-prone subgroups.	IL-33 blockade may be more useful in COPD patients with exacerbation biology than in unselected COPD
Tozorakimab; anti-IL-33				
Maurer et al. (67)	Moderate-to-severe atopic dermatitis	Phase 2 randomized placebo-controlled trial	Astegolimab did not show a significant benefit vs. placebo for primary or secondary outcomes.	IL-33/ST2 may contribute to AD, but blocking this pathway alone is unlikely to control chronic dermatitis broadly
Astegolimab; anti-ST2				
Silverberg et al. (68)	Moderate-to-severe atopic dermatitis	FRONTIER-2 phase 2a randomized study	Primary EASI endpoint was not met; numerical improvements in responder rates and pruritus were seen at higher dose.	In chronic skin disease, IL-33 blockade may need better biomarker selection or combination strategies
Tozorakimab; anti-IL-33				
Corren et al. (71)	Early-onset moderate-to-severe asthma	FRONTIER-3 phase 2a randomized study	Primary endpoint was not met, but efficacy signals were observed in selected patients, especially those with prior exacerbations.	IL-33-targeted therapy may work best when epithelial injury and exacerbation pathways are active
Tozorakimab; anti-IL-33				

In atopic dermatitis, a proof-of-concept study with the anti-IL-33 antibody etokimab showed clinical improvement after a single dose, suggesting that IL-33 blockade can alter disease activity in humans (65). Etokimab also showed activity in a small, randomized study in peanut allergy, where treatment increased antigen tolerance and reduced atopy-related adverse events (66). These early findings were encouraging because they supported IL-33 as an upstream regulator of allergic responses. However, later studies in chronic inflammatory skin disease were less convincing. Astegolimab, an anti-ST2 antibody, did not meet its primary endpoint in moderate-to-severe atopic dermatitis, and tozorakimab also failed to show a statistically significant improvement in the primary EASI endpoint in FRONTIER-2, although some response rates were numerically higher at the highest dose (67, 68). Together, the skin studies suggest that IL-33 may contribute to atopic inflammation, but chronic dermatitis is unlikely to be controlled by IL-33/ST2 blockade alone in unselected patients.

Asthma has provided stronger evidence that the IL-33/ST2 pathway is clinically relevant, although the results remain mixed. Itepekimab, an anti-IL-33 antibody, improved asthma control, lung function, and quality-of-life measures in patients with moderate-to-severe asthma, supporting the idea that IL-33 can drive clinically meaningful airway inflammation (69). Astegolimab, which targets ST2 rather than IL-33 itself, reduced the annualized exacerbation rate in adults with severe asthma and appeared to have activity even in patients with lower eosinophil counts (70). This suggests that IL-33/ST2 blockade may reach beyond classic eosinophilic type 2 disease. More recently, tozorakimab did not meet the primary endpoint in FRONTIER-3, a phase 2a study in early-onset asthma, but showed signals in selected patients, particularly those with prior exacerbations (71). This pattern matters. IL-33 blockade may be more useful in patients whose disease is driven by epithelial stress and exacerbation biology than in broad asthma populations defined only by lung function or diagnostic label.

In a phase 2a trial, itepekimab did not meet the primary endpoint in the overall COPD population, but subgroup analyses showed reductions in exacerbations and improvements in lung function among former smokers. Current smokers did not show the same benefit (72). This difference is biologically plausible, given the oxidative and inflammatory burden of active smoking, which may alter IL-33 release, oxidation, or downstream signaling. Astegolimab also produced mixed results in COPD: it did not significantly reduce exacerbation rate in the COPD-ST2OP trial, but it improved health-status measures compared with placebo (73). Tozorakimab, in the FRONTIER-4 phase 2a COPD study, showed signals suggesting that lung function responses may be enriched in patients with type 2 inflammatory features or exacerbation-prone disease (74). These COPD data do not argue against IL-33 as a target. Rather, they show that the tissue context matters; smoking status, epithelial injury, inflammatory phenotype, and redox state may all influence whether IL-33 blockade is beneficial.

The differences between the treatment methods are also informative. Etokimab and itepekimab neutralize IL-33 itself. Astegolimab blocks ST2 and prevents signaling through the IL-33/ST2 canonical pathway. Tozorakimab was developed with a more mechanistic rationale: it inhibits IL-33 signaling through ST2 and also prevents IL-33-associated RAGE/EGFR signaling linked to epithelial dysfunction (29). This distinction matters because not all IL-33 biology can be captured by blocking ST2 alone. ST2 blockade may inhibit canonical inflammatory signaling, while ligand-directed approaches may intercept a wider set of IL-33-dependent effects. The downside is that a broader neutralization could also interfere with useful IL-33 functions, including those involved in epithelial adaptation and repair.

Most IL-33/ST2 trials have not directly measured tissue repair as a primary biological outcome. They have focused on exacerbations, lung function, symptom scores, EASI responses, and safety. These endpoints are important, but they do not indicate whether IL-33 blockade preserves or impairs repair following epithelial injury. Therefore, the evidence for IL-33 as a reparative pathway is currently much stronger in preclinical systems than in human trials. Murine injury models and epithelial regeneration studies show that controlled IL-33 signaling can promote tissue protection through ILC2- and Treg-associated amphiregulin pathways (25, 43–45). More recent work has also identified a tissue-intrinsic IL-33/EGF circuit that supports epithelial regeneration after intestinal injury, including evidence from organoid-based systems (75). However, comparable repair-focused endpoints have not been incorporated into most clinical trials of IL-33/ST2 blockade. Thus, the current clinical evidence supports IL-33/ST2 blockade primarily as an anti-inflammatory strategy, while its effects on epithelial repair remain largely inferred rather than directly tested.

The mixed clinical outcomes also highlight the need for better biomarkers. Blood eosinophils may help in some settings, but they are unlikely to capture the full biology of IL-33. Smoking status, exacerbation history, epithelial injury signatures, circulating sST2, IL-33 redox state, and markers of airway remodeling may all be relevant for identifying patients most likely to benefit. The COPD experience is particularly instructive; the difference between former and current smokers in the itepekimab trial suggests that the tissue environment may determine whether IL-33 blockade works (72). Similarly, the asthma and dermatitis studies suggest that the disease label alone is not enough. The pathway must be active in the right compartment, at the right time, and in the right inflammatory state.

Future therapeutic methods should therefore move away from the idea of simply “turning off” IL-33. A more realistic goal is to narrow the pathogenic signaling window while preserving short-lived, localized IL-33 responses that contribute to tissue adaptation. This may require selecting patients based on epithelial injury or exacerbation biology, distinguishing acute IL-33 release from chronic proteolytic amplification and incorporating biomarkers that reflect both inflammation and repair. Clinical trials so far have taught a consistent lesson: IL-33 can be therapeutically targeted, but blockade is most likely to succeed when treatment is matched to the biological context driving the disease.

## 8 Discussion

This review proposes a different way of thinking about IL-33. Instead of viewing IL-33 only as a cytokine released after tissue damage, we follow it through a sequence of regulated states. IL-33 starts in the nucleus, where it is stable and restrained. It then becomes available exclusively for release under certain conditions of stress, injury or barrier disruption. Once outside the cell, IL-33 is further modified by proteases, oxidation, receptor availability and the tissue's metabolic state. The final biological outcome is determined by all these steps.

**Figure 1:**
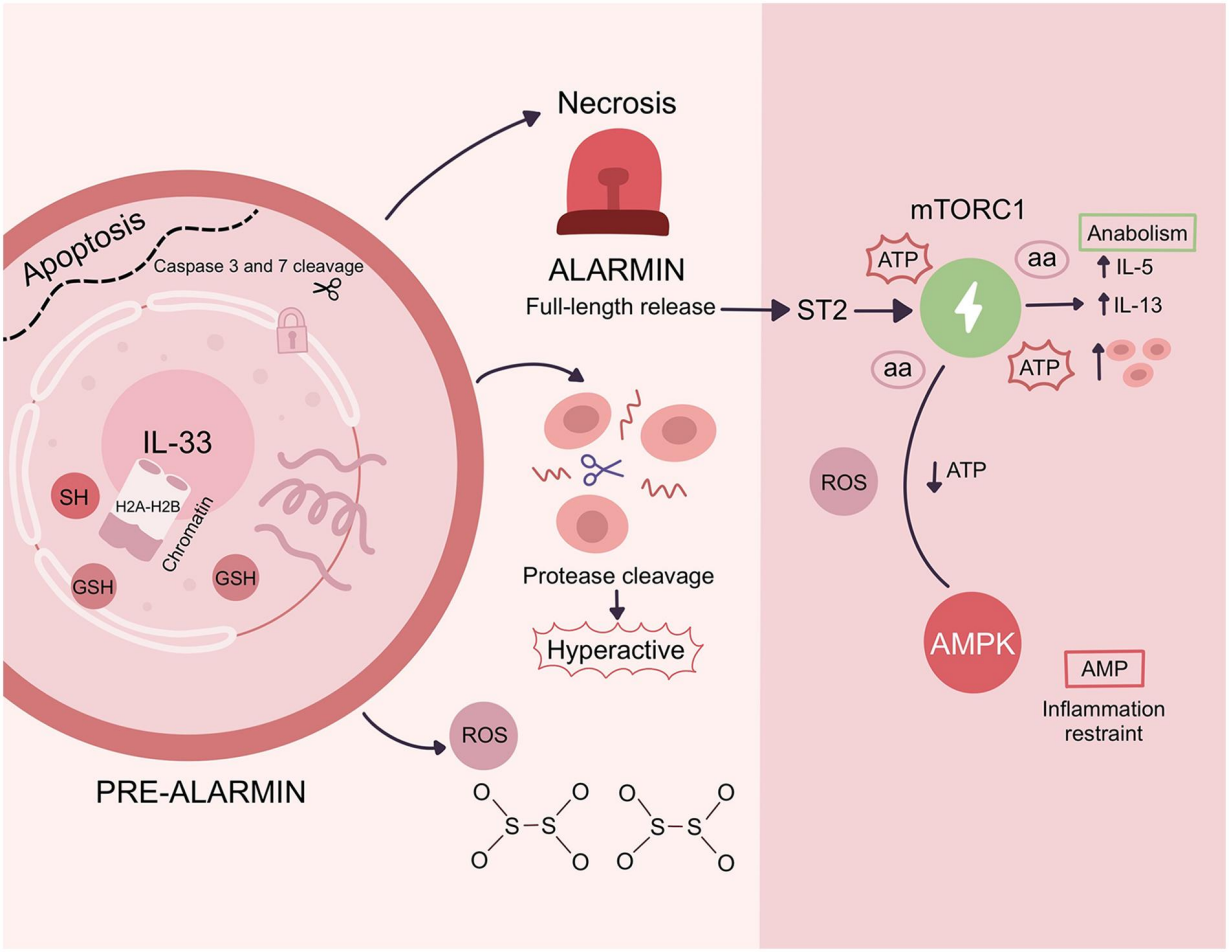
Conceptual summary of IL-33 as a checkpoint-regulated alarmin. Under homeostatic conditions, IL-33 remains in the nucleus of structural cells and is bound to chromatin (H2A-H2B). When necrotic damage or protease-driven stress occurs, IL-33 is released into the extracellular space as an intact full-length or cleaved active protein. Under apoptotic or oxidative conditions, IL-33 is inactivated by caspases or by forming disulfide bonds. In a protease-rich tissue environment (e.g., neutrophil elastase, cathepsin G, mast cell chymase, or environmental proteases from allergens), IL-33 is proteolytically trimmed into shorter “mature” forms that are active and induce IL-33 receptor/ST2 signaling. IL-33 activates ST2, initiating the PI3K-AKT-mTORC1 signaling pathway. These pathways boost glycolysis, lipid production, and cytokine production and release. When the AMP-activated protein kinase (AMPK) is activated in response to metabolic or oxidative stress, it reduces inflammation and helps restore cellular balance.

A major contribution of this review is the concept of the pre-alarmin phase. This term helps to separate nuclear IL-33 from extracellular IL-33. Nuclear IL-33 is not already working as an alarmin, but it is also not irrelevant. It is present, protected, and ready to be mobilized. This frame shifts attention from IL-33 release as a single event to the mechanisms that determine whether release occurs, what form IL-33 takes and how strongly it signals afterward. In this sense, nuclear retention is not just a cell-biological detail. It is the first checkpoint in the IL-33 signaling (Figure 1).

Intrinsic regulation controls IL-33 release from the cell. It includes transcriptional priming, the mode of cell death, epithelial stress pathways and regulation of release. Extrinsic regulation controls events that occur after IL-33 release. It involves proteolytic amplification, oxidative inactivation and receptor competition. This distinction is useful because release and activation are often discussed together even though they are mechanistically different. A stimulus may release IL-33 without strongly increasing its activity. In contrast, a protease-rich environment can amplify the inflammatory signal from released IL-33.

Another point is that repair and inflammation are not separate stories in the IL-33 biology. IL-33 is often discussed as a driver of type 2 inflammation, particularly in asthma, allergy and chronic airway disease. Yet, the same pathway can also support epithelial repair, tissue protection and regulatory immune programs. The difference seems to depend on timing, location and intensity. A short, localized IL-33 signal may help restore tissue stability. A prolonged or amplified signal can maintain inflammation, remodeling and fibrosis. Thus, repair and pathology can represent different outcomes of the same pathway under different regulatory conditions.

The metabolic layer also adds another important dimension. The IL-33-driven responses require energy. Cytokine production, immune cell expansion, amphiregulin release and epithelial restoration all depend on metabolic resources. IL-33 signaling is therefore formed not only by damage, but also by the metabolic capacity of the tissue to respond to this damage. This supports the idea that IL-33 regulates inflammatory permissiveness. IL-33 responses may proceed when energy and redox conditions permit, but they may be limited or redirected under metabolic stress.

Trials targeting IL-33 or ST2 have demonstrated potential in asthma, COPD, atopic dermatitis and allergy, but the results have not been uniform (Table 2). This variation does not mean that the path is unimportant. Instead it suggests that IL-33-targeted therapy will require patient selection. The disease label alone is not sufficient. A patient with asthma, COPD or dermatitis may or may not have active IL-33 biology at the time of treatment. Smoking status, epithelial injury, exacerbation history, protease-rich inflammation, redox state and biomarkers such as sST2 may all matter.

A major gap in the clinical literature is that repair has rarely been measured directly. Most IL-33/ST2 trials have focused on symptoms, exacerbations, lung function, EASI scores, or safety. These are important endpoints, but they do not show whether IL-33 blockade affects epithelial restoration after injury. This gap matters because preclinical studies suggest that IL-33 can support tissue repair through ILC2- and Treg-associated amphiregulin pathways. Future trials should therefore ask not only whether IL-33 blockade reduces inflammation, but also whether it preserves the short-lived repair responses needed after epithelial damage.

Despite these advances, several important gaps remain. Nuclear retention is clearly important for limiting inappropriate extracellular activity, but whether nuclear IL-33 also acts as a broad and physiologically relevant transcriptional regulator remains unresolved. The available data suggests that this may depend on cell type, inflammatory state, and experimental context. A second gap is the clinical use of IL-33-related biomarkers. Circulating sST2, epithelial injury signatures, IL-33 oxidation state, eosinophils, and exacerbation history may each reflect part of the pathway, but no single biomarker currently defines IL-33-dependent disease. Finally, patient stratification remains a major challenge for IL-33-targeted therapy. The mixed clinical trial results suggest that disease labels such as asthma, COPD, or atopic dermatitis are insufficient by themselves. Future studies will need to identify when the IL-33/ST2 axis is active, in which tissue compartment, and whether blockade is likely to reduce pathogenic inflammation without compromising repair.

Overall, this review presents IL-33 as a checkpoint-regulated alarmin. Its biology starts in the nucleus, continues through regulated release, is edited in the extracellular space and is determined by tissue metabolism. This model helps explain why IL-33 can promote inflammation in one setting and repair damage in another. It also provides a rationale for more precise clinical interventions. The goal should not be to suppress IL-33 indiscriminately, but to understand when its signal becomes pathogenic and how to limit it while preserving its homeostatic and reparative functions.
